# Potential alternatives in treatment of kawasaki disease to reduce the extremely rare risk of reye syndrome

**DOI:** 10.3389/fped.2026.1856121

**Published:** 2026-09-08

**Authors:** Ethan J. Johnson, Jordan D. Moser, Benjamin S. Buehrer, Khiet Ngo, Rupesh Raina

**Affiliations:** 1 College of Medicine, Northeast Ohio Medical University, Rootstown, OH, United States; 2 College of Medicine and Health Sciences, University of North Dakota, Grand Forks, ND, United States; 3 Department of Nephrology, Akron Nephrology Associates/Cleveland Clinic Akron General Medical Center, Akron, OH, United States

**Keywords:** aspirin, cardiology, hematology, Kawasaki disease, pediatrics, Reye syndrome, vasculitis

## Abstract

**Background/objective**: Kawasaki disease is an acute, systemic vasculitis most commonly observed in young Asian children. While the exact pathogenesis remains unknown, it is thought to be triggered by a pathogenic exposure that elicits an inflammatory response several days to 2 weeks before the onset of symptoms. Symptoms include edematous hands and feet, conjunctivitis, mucositis, a diffuse erythematous rash, lymphadenopathy, and a fever lasting for at least 5 days, which is required for diagnosis. In addition, coronary vessels may exhibit aneurysms, thrombosis, and stenosis. Without treatment, Kawasaki disease carries a 25% risk of developing coronary aneurysms. However, with the timely administration of intravenous immunoglobulin, this risk is reduced to 4%. Given the inflammation and possible thrombosis of the coronary vasculature, current guidelines recommend aspirin for the management of Kawasaki disease. Kawasaki disease is one of the few conditions in which administering aspirin to children is considered acceptable, despite the risk of inducing Reye syndrome, a potentially fatal condition triggered by a viral infection while taking salicylates such as aspirin. Therefore, this review addresses a key question: why continue to use aspirin with its known risk of Reye syndrome when alternative antithrombotic drugs used in pediatric patients are available? This review seeks to explore potential substitutes for aspirin to mitigate the risk of Reye syndrome while also providing the necessary anticoagulation in Kawasaki disease.

## 1 Introduction

Kawasaki disease (KD) is an acute, systemic vasculitis that primarily affects medium-sized arteries in children under 5 years of age (1). Children of Asian descent exhibit the highest incidence rates, with countries such as Japan and Korea reporting more than 200 cases per 100,000 children under 5 (2). Without treatment, ∼25% of KD patients develop coronary aneurysms (3). This risk is reduced to ∼4% when intravenous immunoglobulin (IVIG) is administered within 10 days of symptom onset. To prevent thrombus formation, patients are also administered aspirin. Step-up therapy for resistant disease includes immunosuppressants such as prednisolone, infliximab, and cyclosporine (4). KD carries few long-term complications in the setting of timely treatment, with a mortality rate of ∼0.2%.

The use of high-dose aspirin in the acute phase of KD has been reported to lead to Reye syndrome, a rare yet serious and potentially fatal condition (5–7). Reye syndrome most commonly occurs when parents mistakenly give aspirin to their children in the setting of a viral illness, predominantly influenza and varicella (4). Reye syndrome is characterized by acute hepatic failure, cerebral edema, encephalopathy, coma, and, if not promptly recognized and treated, death, carrying a mortality rate of ∼20%. Among survivors, ∼33% experience long-term neurological deficits. While the development of Reye syndrome in patients undergoing KD treatment is rare, this review seeks to describe aspirin alternatives for pediatric patients to maintain essential antithrombotic therapy while minimizing the risk of Reye syndrome.

## 2 Background

### 2.1 Pathophysiology of Kawasaki disease

The precise cascade of events that elicits KD is poorly understood; however, it is most likely virally triggered, given its seasonal pattern, which aligns with common pathogens such as influenza during the winter and spring (8). Furthermore, children younger than 6 months rarely develop KD, likely due to passive immunity from maternal antibodies (9). In addition to viruses, researchers have investigated fungi and pesticides as potential triggers for the KD cascade; however, studies have failed to establish a meaningfully link between these sources and the disease (10).

Recent literature has described potential genetic variants that may increase the likelihood of developing KD. For example, inositol 1,4,5-triphosphate 3-kinase C (ITPKC) is a kinase that phosphorylates the signaling molecule of inositol 1,4,5-triphosphate (IP_3_). During T-cell receptor stimulation in an immune response, IP_3_ is released, triggering a cascade of events that lead to T-cell activation and the elicitation of inflammation. Specifically in KD, IL-1β and IL-18 are believed to be the main cytokines driving vascular complications. ITPKC helps regulate this process by limiting IP_3_-mediated signaling, thereby minimizing inflammation (11). However, a functional single-nucleotide polymorphism (SNP) in ITPKC gene has been associated with an increased risk of coronary artery abnormalities in Taiwanese, Japanese, and American patients with KD (11, 12). This increased risk is explained by an improperly functioning ITPKC protein that leads to an exaggerated inflammatory response involving the calcium–nuclear factor of activated T cells (NFAT) pathway (13).

**Figure 1:**
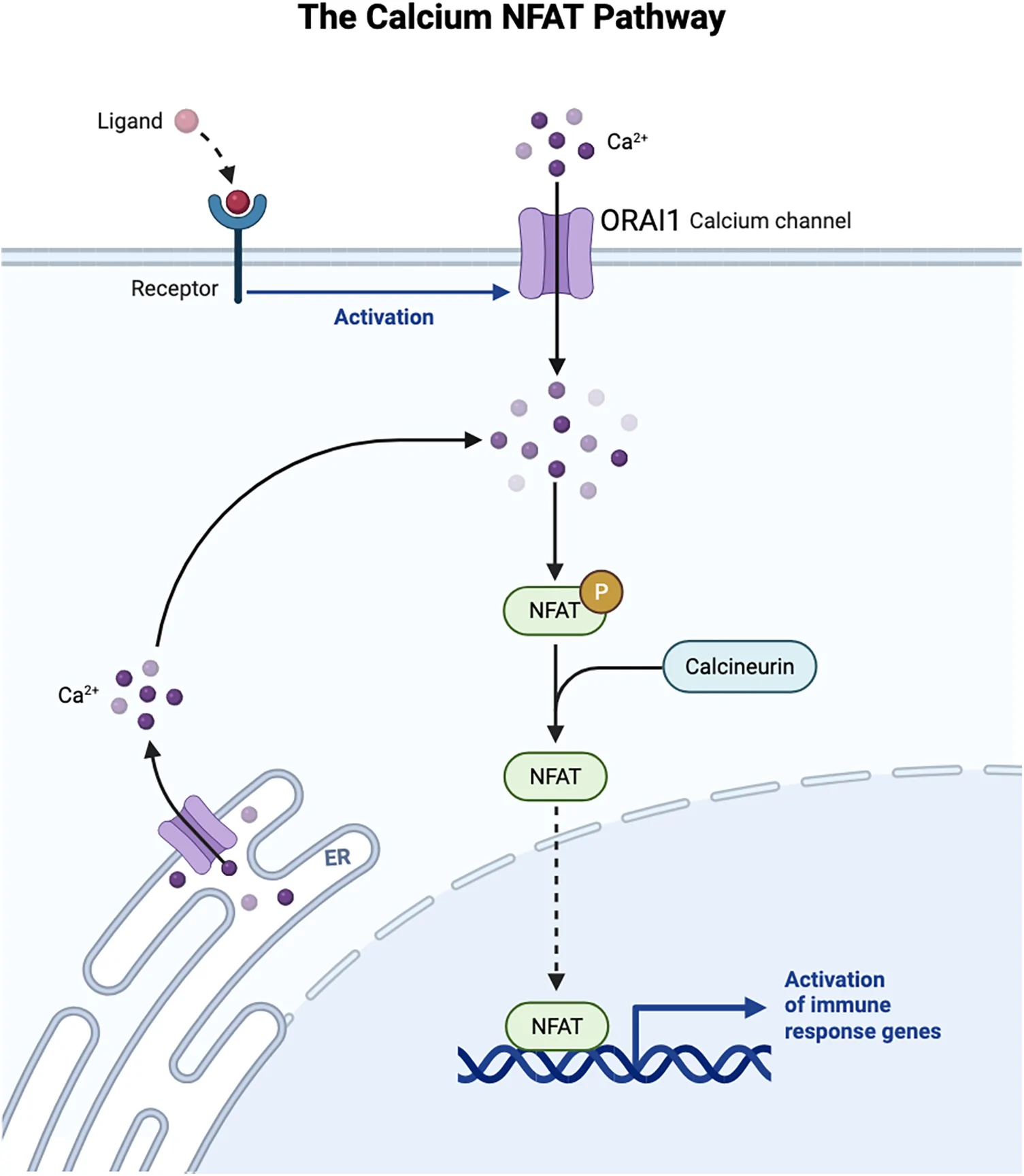
Simplified overview of the calcium-NFAT pathway. Created in BioRender. Johnson, E (2026).

Other notable proteins involved in the calcium–NFAT pathway (Figure 1) impacted by SNPs include the calcium release-activated calcium modulator 1 (ORAI1) calcium channel protein and the Na^+^–Ca^2+^ bidirectional exchanger protein also known as NXC1. An SNP in the ORAI1 protein is associated with KD susceptibility; notably, this mutated protein is 20 times more prevalent in Japan than in Europe (14). An SNP in the sodium–calcium exchanger (NCX1) protein, which is notably expressed in macrophages, has been found in inflammatory cells from aneurysmal wall biopsies, suggesting another genetic predisposition to the development of KD (15, 16).

From a cellular perspective, the first step in the pathology of KD is the activation of the innate immune system, followed by neutrophilic infiltration of the tunica intima, tunica media, and part of the arterial adventitia (17). Neutrophils then release inflammatory proteins, notably alarmins of the S100 protein family that induce vascular damage. Significantly, S100 proteins such as calprotectin are found in markedly higher concentrations during the acute phase of KD than in other viral diseases. Calprotectin levels are also elevated in patients with coronary aneurysms, highlighting its crucial role in the formation of coronary aneurysms in KD (18). Regarding the adaptive immune system, the literature has proposed a wide range of mechanisms that often contradict one another, but overall, the pathophysiology of KD is becoming clearer over time. Ultimately, there are many components of KD from the innate and adaptive immune systems, along with potential genetic influences, that offer an abundance of therapeutic targets for managing KD (9). By understanding these genetic predispositions to KD, future therapies could potentially prevent KD from developing altogether, thereby eliminating the potential, rare risk of Reye syndrome.

### 2.2 Pathophysiology of Reye syndrome

In contrast to Kawasaki disease, the pathophysiology of Reye syndrome is more clearly characterized, although not yet fully elucidated. Reye syndrome is strongly associated with aspirin exposure during viral illnesses in children and is thought to involve mitochondrial dysfunction leading to acute encephalopathy and hepatic injury. Specifically, this mostly happens when parents provide their children aspirin to relieve their child's symptoms while remaining unaware of its side effects or inadvertently provide a drug containing aspirin, such as Excedrin.

After ingestion, aspirin is rapidly absorbed primarily in the stomach and small intestine, undergoing deacetylation from acetylsalicylic acid to salicylic acid. After absorption, aspirin undergoes first-order kinetic elimination in the liver with a half-life of 2–4.5 h (19). The mechanism of action of aspirin involves irreversibly inhibiting cyclooxygenase 1 (COX-1) and cyclooxygenase 2 (COX-2). By inhibiting COX-1, arachidonic acid cannot be converted into thromboxane A2 (TXA_2_) and prostaglandins. The main role of TXA_2_ is to stimulate the thromboxane prostanoid receptor, a G-protein-coupled receptor, to promote GPIIbIIIa receptors for platelet aggregation, changes in platelet shape for efficient plaque formation, vasoconstriction of smooth muscle cells, and other prothrombotic effects (20). COX-2 inhibition prevents a multitude of inflammatory pathways from being activated, notably by inhibiting NF-*κ*B, a potent transcription factor responsible for initiating the synthesis of cytokines such as TNF-α, IL-1, and IL-6. COX-2 inhibition also leads to decreased levels of C-reactive protein, a potent inflammatory protein that plays a significant role in the innate immune system (21).

Due to the inhibition of both COX pathways, aspirin serves as an antiplatelet and anti-inflammatory agent in KD. However, while aspirin may reduce the risk of myocardial infarction, it brings the exceptionally rare risk of Reye syndrome. The main mechanism of Reye syndrome involves the breakdown of salicylic acid, the active metabolite of aspirin, by hepatocytes. First, salicylic acid enters the mitochondrial matrix from the cytoplasm of hepatocytes via passive diffusion. Then, it is metabolized to salicyl-CoA via the acyl-CoA synthetase medium-chain family member 2B (ACSM2B) enzyme. Salicyl-CoA then undergoes phase 2 metabolism by conjugation with glycine via glycine N-acyltransferase, where it is converted to salicyluric acid to be excreted by the kidneys (22).

KD patients often have concurrent viral illnesses, including influenza, varicella, adenovirus, hepatitis A, and others. These viral illnesses make patients prone to hepatic inefficiency, leading to an accumulation of salicylic acid within hepatocytes. Poor metabolism of salicylic acid inhibits the activity of the trifunctional mitochondrial enzyme 3-hydroxyacylCoA dehydrogenase, thereby contributing to impaired beta-oxidation of fatty acids. Defective fatty acid metabolism contributes to reduced gluconeogenesis, elevated fatty acids, and increased ammonia, leading to fatty degeneration of the liver and encephalopathy (23).

As KD is likely a collection of multiple components that promote the disease, multiple factors may contribute to the likelihood of developing Reye syndrome. Besides viral illnesses, metabolic diseases, such as medium-chain acyl-CoA dehydrogenase deficiency, increase the risk of Reye syndrome by impairing the ability of the patient to process fatty acids (24). Other medications may also increase the risk of Reye syndrome, such as valproic acid, which, due to its fatty acid structure, can interfere with carnitine palmitoyltransferase I. This enzymatic interference may lead to an attenuated response in hepatic metabolism in children with normal hepatic function prior to receiving valproic acid (25). Awareness of these additional factors offers a better opportunity for successful treatment and recovery.

Therefore, Reye syndrome can quickly lead to death if not treated properly. However, myocardial infarction due to a potential coronary aneurysm in KD poses a serious and much more prominent threat to the well-being of KD patients compared to Reye syndrome. To help minimize the risk of a myocardial infarction while also avoiding Reye syndrome, alternative antithrombotic drugs could offer a potential solution.

## 3 Pharmaceuticals

### 3.1 Antiplatelet and anticoagulant agents

**Figure 2:**
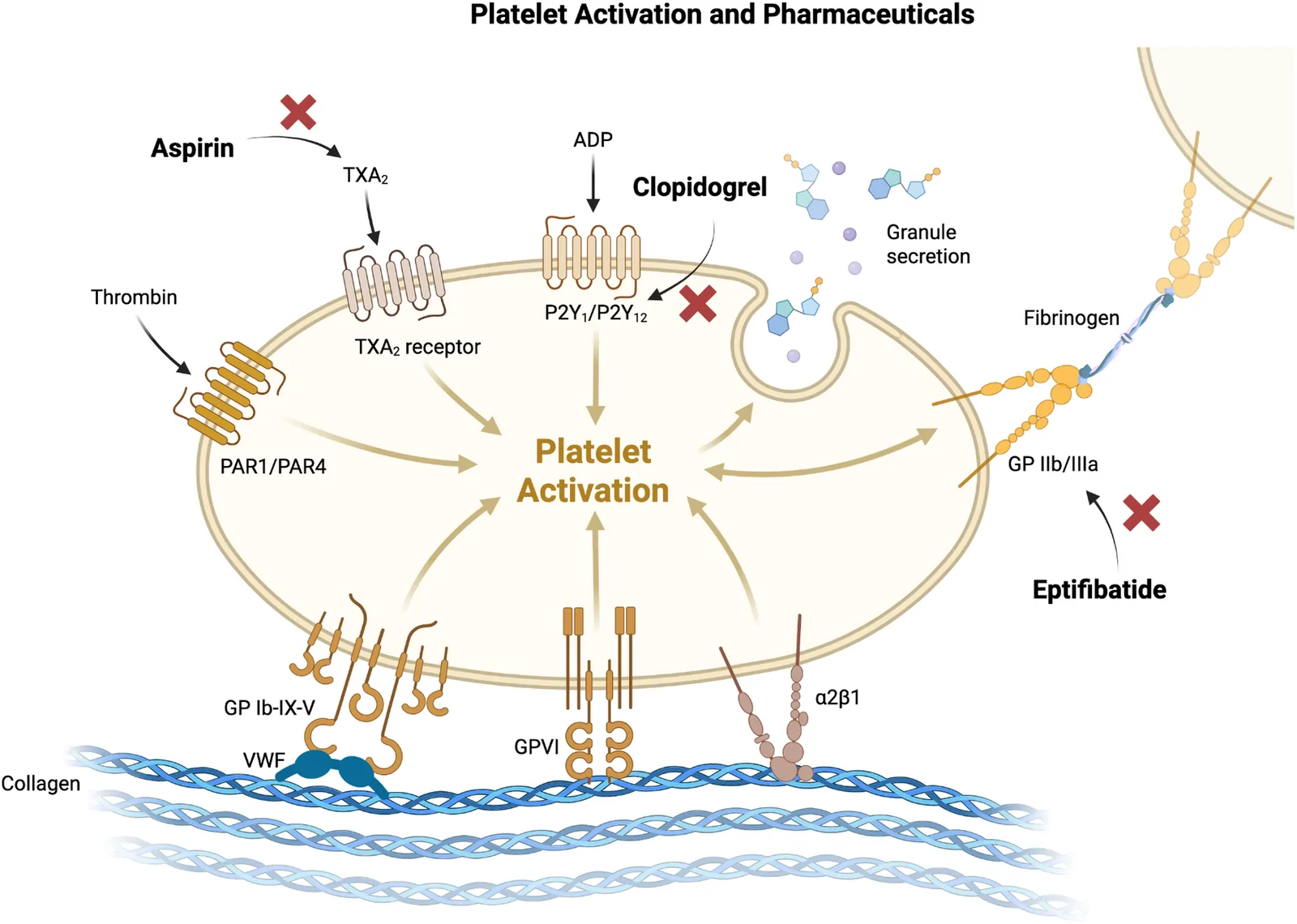
Platelet activation and simple overview of several antiplatelet drugs including aspirin, clopidogrel and eptifibatide. Created in BioRender. Johnson, E. (2026).

Aspirin is considered an antiplatelet agent or a drug that inhibits platelet activation and aggregation (26). Several other classes of antiplatelets exist that act through various mechanisms, including drugs such as clopidogrel and ticagrelor, which act as antagonists of the P2Y12 receptor to prevent adenosine diphosphate-mediated activation of platelets. Glycoprotein IIb/IIIa inhibitors such as eptifibatide or abciximab function by preventing fibrinogen from binding the glycoprotein IIb/IIIa receptor, thereby preventing platelet aggregation (Figure 2). Finally, phosphodiesterase inhibitors such as cilostazol increase cyclic adenosine monophosphate by inhibiting phosphodiesterase 3, thereby preventing platelet activation.

What makes aspirin unique is its anti-inflammatory properties. As noted above, aspirin irreversibly inhibits COX enzymes via acetylation, so that inflammatory prostaglandins, transcription factors, and cytokines are not produced. The clinical relevance of the anti-inflammatory capabilities of aspirin varies. Depending on the patient population and the inflammatory pathway affected, the literature regarding the anti-inflammatory effect of aspirin may be moderate or substantial (27, 28). Because the pathophysiology of KD is driven by inflammation leading to coronary aneurysms and other abnormalities, aspirin is vital in protecting against these adverse coronary changes. If coronary abnormalities do develop, aspirin provides antithrombotic therapy to minimize the risk of clot formation. IVIG is also believed to play a crucial role in minimizing inflammation by suppressing both the adaptive and innate immune systems in multiple ways (29). As discussed, the effectiveness of aspirin in reducing inflammation in KD may warrant further investigation, with recent literature suggesting that IVIG and steroids play a more prominent role in anti-inflammation therapy.

**Figure 3:**
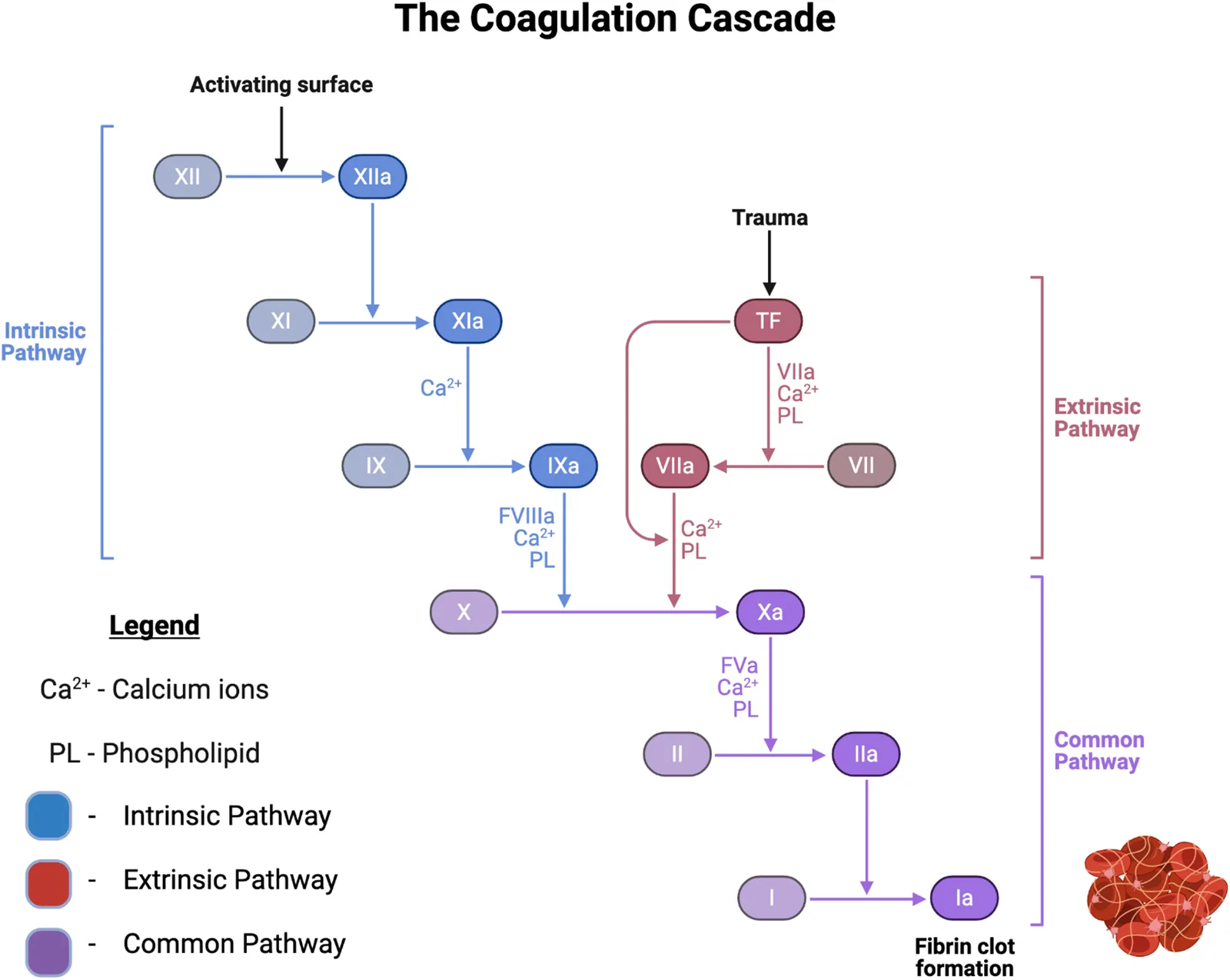
The coagulation cascade, a biochemical process of forming a hemostatic plug with a variety of targets of which pharmaceuticals may act. Created in BioRender. Johnson, E. (2026).

Besides antiplatelets, anticoagulant agents are another major group of antithrombotics that inhibit the coagulation cascade. The primary goal of inhibiting the coagulation cascade is to prevent the conversion of fibrinogen to fibrin, thereby preventing cross-linking and clot stabilization. Examples of anticoagulants include heparin, enoxaparin, warfarin, dabigatran, apixaban, bivalirudin, and many others, with each of the previously listed having a unique mechanism of action through the many targets of the coagulation cascade (Figure 3) (20).

### 3.2 Clinical application

Among children under 5 years of age, the incidence rate of KD is 265 cases per 100,000 in Japan vs. 19 per 100,000 children in the United States (30). The difference in incidence may be attributable to genetic variations between children of Asian and European descent, as discussed above.

Reye syndrome has a much lower incidence rate than KD. Since 1994, the United States has reported fewer than two cases annually. A major contributor to such a low incidence rate is the issuance of warnings to the general public about the risk of Reye syndrome from the use of salicylates in children with viral infections. In the 1970s, surveillance for Reye syndrome was started (31). A total of 555 cases of Reye syndrome were reported in 1980, which led to the initiation of issuance of warnings about Reye syndrome from the use of salicylates. By 1987, 36 cases were reported yearly, and this rate declined to the current rate of fewer than 2 yearly (32).

The development of Reye syndrome secondary to aspirin treatment is exceedingly rare. Currently, no studies have analyzed the incidence of Reye syndrome associated with therapeutic aspirin treatment for KD. Nevertheless, the risk is certainly real, as documented by many case reports (5–7, 33, 34). However, considering that the incidence of KD hospitalizations in the United States is 18–20 per 100,000 children, with ∼25% of untreated patients experiencing major adverse cardiac event (MACE), the need for prompt KD therapy is undeniable and far outweighs the risk of Reye syndrome.

The clinical use of aspirin falls into acute and chronic phases (1). In the acute phase of KD, which typically lasts 10–14 days, IVIG at 2 g/kg is administered as a single infusion over 8–12 h. IVIG is crucial in the treatment of KD because it reduces the risk of developing coronary abnormalities to less than 5% (29). This effect may or may not be amplified not through aspirin but rather by steroids, such as methylprednisolone, as several studies have found the use of steroids to significantly improve outcomes for patients with KD (35). In addition, high-dose aspirin, 20–25 mg/kg, is administered every 6 h for a total of 80–100 mg/kg/day. The duration of high-dose aspirin varies across health systems, but many switched to low-dose aspirin after they had been afebrile for 48–72 h. Low-dose aspirin, given at 3–5 mg/kg orally once daily, is then continued until the patient has no evidence of coronary changes 6–8 weeks after the onset of KD, which may be confirmed by echocardiography. Of note, for children who do develop coronary abnormalities, aspirin may be continued indefinitely, theoretically putting the patient at a constant risk of Reye syndrome.

The previously discussed dosing regimen is currently used in the United States. The aspirin regimen for KD varies slightly in China, Japan, and parts of Europe, where a medium-dose regimen of 30–50 mg/kg per day is used to reduce the risk of adverse effects associated with high-dose aspirin, including bleeding, gastrointestinal ulcers, urticaria, and, although rare, Reye syndrome (1, 36). Furthermore, there is no evidence that high-dose aspirin is superior to medium-dose aspirin (1, 37). In addition, an andomized control trial (RCT) comparing high-dose aspirin plus IVIG with IVIG alone among 134 children with KD showed no statistical difference between the groups regarding the incidence of coronary artery abnormalities. The aspirin plus IVIG group exhibited a reduction in coronary abnormalities from 13.0% to 2.9% at 6 weeks and 1.4% at 6 months compared with the IVIG-only group, which went from 10.8% to 1.5% at 6 weeks and 3.1% at 6 months, ultimately showing no significant difference between the groups regarding the frequency of coronary abnormalities [0.7 percentage points (95% CI, −4.5 to 5.8 percentage points); *P* = 0.65] (38). Reinforcing the value of IVIG, a recent meta-analysis concluded that when patients are properly treated with IVIG, there is no significant difference in the incidence of coronary abnormalities, fever duration, and hospital stay whether high-dose aspirin and no- or low-dose aspirin is used (39).

Furthermore, corticosteroids have been shown to improve outcomes in the treatment of KD. The RAISE trial, an RCT involving 248 KD patients in Japan, reinforced this finding by assigning 123 patients to a control group receiving IVIG (2 g/kg over 24 h) and aspirin (30 mg/kg/day), followed by low-dose aspirin (3–5 mg/kg/day) for at least 28 days after fever onset. This group was then compared with a group of 125 patients who received the same IVIG dosing as the control group, augmented with prednisolone (2 mg/kg/day), tapered according to the condition of the patient. Overall, the incidence of coronary artery abnormalities was significantly lower in the treatment group (4 patients) than in the control group (28 patients) (*P*-value <0.001) over a 2-year period (40).

For KD treatment, this study demonstrates that steroids may be more effective at preventing coronary abnormalities than aspirin. What the RAISE trial does not account for is the antiplatelet properties of aspirin. Corticosteroids are primarily used for their anti-inflammatory rather than antiplatelet properties. Other studies, including a partially retrospective study based on the original RAISE trial, reported different findings regarding the effectiveness of corticosteroids in KD. In a cohort of 221 North American children with high-risk KD, 83 were actively treated with a “modified RAISE” or “modified RAISE (mRAISE)” regimen of 2 mg/kg/day of IV methylprednisolone for 5 days, followed by oral prednisone (2 mg/kg/day for 5 days with a subsequent taper over 10 days). A total of 136 patients were retrospectively analyzed in the “non-mRAISE” group, either receiving no corticosteroids (82 patients) or receiving corticosteroids as part of a non-mRAISE regimen (56 patients).

To focus on more discrete parameters, this review considers only the 82 patients in the mRAISE group who did not receive corticosteroids, for whom the mRAISE group showed no significant difference in the primary outcome (*P* = 0.50) (41). Of note, the mRAISE group was not treated with aspirin, providing evidence that aspirin may indeed be required to meaningfully reduce the formation of coronary abnormalities.

Therefore, with conflicting evidence regarding the effectiveness of anti-inflammatory properties of aspirin in KD treatment, along with the rare risk of Reye syndrome associated with aspirin, studies analyzing alternatives to aspirin to ensure proper antithrombotic therapy for KD patients are warranted. This is especially important considering that corticosteroids are known for their anti-inflammatory properties, not their antiplatelet properties, which, when considered, are secondary to their anti-inflammatory actions. Therefore, if the inflammatory response in KD is truly quelled by IVIG, steroids, or a combination of the two, then the antiplatelet quality of aspirin warrants further study.

Unfortunately, RCTs comparing antiplatelet agents to aspirin in the management of KD are severely limited due to the low patient population and even the smaller percentage who experience thrombotic events. This leads to the use of retrospective studies, and Gao et al. analyzed the use of clopidogrel vs. aspirin in patients with Kawasaki disease (42). Evaluating liver injury via alanine transaminase (ALT) and total bilirubin levels, this study reviewed 66 children treated with aspirin and 100 children treated with clopidogrel, both in combination with standard IVIG treatments, between June 2020 and December 2021 in China.

After analyzing posttreatment ALT values, children treated with aspirin were found to have persistently higher ALT levels (19.7%) compared with the clopidogrel group (2.0%), with a *P*-value less than 0.001. Furthermore, the non-response rates to IVIG treatment were significantly lower (*P* = 0.030) in the clopidogrel group (29.0%) than in the aspirin group (45.5%).

This study concluded that clopidogrel may be an alternative antiplatelet agent that causes less hepatic damage than aspirin. This should not be surprising, given that hepatoxicity from clopidogrel is rare (43). Of note, this study had a small number of participants and was compromised by selection bias due to its unbalanced, non-randomized groups. Confounding by indication also presents a limitation with this study; because the treatment groups were unbalanced, outcome differences may stem from sicker KD patients rather than drug effects. Furthermore, this was a retrospective observational study, not an RCT. Aside from this study, there are no other obvious, reliable studies that compare aspirin and clopidogrel. However, Li et al. proposed an abstract and design for a potential randomized controlled trial comparing aspirin alone vs. aspirin plus clopidogrel in KD patients, although the trial has yet to be conducted (44). Therefore, caution must be exercised when reflecting on the study by Gao et al., given the lack of RCTs in this aspect of KD medicine. Despite being a retrospective observational study, this research provides a foundation for antithrombotic comparisons with aspirin, contributing to an aspect of medicine that is markedly deficient in the literature.

Despite the limited number of clinical trials analyzing the use of clopidogrel in children, current guidelines recommend clopidogrel combined with aspirin, depending on the coronary status of KD patients (1, 3). What they do not recommend is using clopidogrel as first-line prophylactic antithrombotic treatment, despite evidence showing that monotherapy with clopidogrel is superior to aspirin in preventing major adverse cardiovascular or cerebrovascular events (MACCE) across seven randomized trials involving 28,982 adult patients (45).

Specifically, the SMART-CHOICE 3 trial, a multicenter, randomized, open-label trial of 5,506 adult patients, further emphasized clopidogrel's safety by comparing it with aspirin monotherapy in patients who had completed dual antiplatelet therapy following percutaneous coronary intervention with drug-eluting stents (46). Over a period of nearly 3 years, this study found that clopidogrel significantly reduced the risk of death from MACCEs, specifically a “composite of all-cause mortality,” myocardial infarction (MI), or stroke. Of these patients, events occurred in 92 patients [4.4% (3.4–5.4)] in the clopidogrel group vs. 128 patients [6.6% (5.4–7.8)] in the aspirin group, yielding a hazard ratio (HR) (95% CI) of 0.71 (0.54–0.93), *P* = 0.013.

When comparing drugs to aspirin, the risk of causing an adverse event must be considered. For antithrombotic agents, the main adverse event of concern is bleeding. The SMART-CHOICE 3 trial compared the risk of type 2, 3, and 5 bleeding events, as defined by the Bleeding Academic Research Consortium (BARC), with types 1 and 4 representing non-actionable bleeding and CABG bleeding, respectively. Over the 3 years, there was no significant difference in bleeding risk between clopidogrel and aspirin [3.0% (2.0–3.0) vs. 3.0% (2.2–3.9); hazard ratio=0.97 (0.67–1.42)]. Therefore, while trials such as the SMART-CHOICE 3 study provide insights into clopidogrel use, they were conducted exclusively in *adult* patients rather than *pediatric* patients, representing a significant difference in patient populations. Coronary vasculature differs significantly between adult and pediatric patients, considering how adult vasculature has had decades of exposure to risk factors such as hypertension and hyperlipidemia. Therefore, RCTs involving pediatric rather than adult populations are required to determine drug efficacy and safety in KD management.

However, it should be noted that none of these clopidogrel trials, aside from Gao (2023), included pediatric patients. One reason the FDA has not approved the use of clopidogrel in children is the CLARINET trial, a multicenter, randomized, event-driven trial that compared clopidogrel with standard therapy, primarily aspirin (47). Specifically, these patients were infants with cyanotic congenital heart disease treated with a systemic-to-pulmonary-artery shunt. After following 906 patients for up to 12 months to assess a “primary efficacy end point,” including but not limited to shunt thrombosis, no significant difference was found. Among these, 89 patients in the clopidogrel group (19.1%) and 90 in the placebo group (20.5%) experienced a primary end point, yielding an absolute risk reduction of 1.4% and a relative risk reduction with clopidogrel of 11.1% (95% CI –19.2 to 33.6; *P* = 0.43). Of note, both clopidogrel and placebo groups had similar bleeding rates (18.8% and 20.2%, respectively).

Therefore, while the CLARINET trial showed no benefit of clopidogrel over aspirin, the patient population differed significantly from that of standard KD patients. This fact, along with how there has only been one retrospective observational study comparing clopidogrel to aspirin among KD patients, calls for further investigation. If clopidogrel is not used, drugs such as heparin, low-molecular-weight heparin (LMWH), and vitamin K antagonists (VKAs) may be considered, given their established use in other hypercoagulable pediatric conditions (48). However, the primary focus should be on antiplatelet agents, as anticoagulant agents are primarily used for venous thrombosis prevention, not arterial thrombosis prevention. While anticoagulants are an unlikely fit for KD therapy, all aspects of potential treatment call for investigation, including the relatively new direct oral anticoagulants (DOACs).

### 3.3 Direct oral anticoagulant trials

Children diagnosed with KD who develop large or giant coronary artery aneurysms (CAAs) face an increased risk of coronary thrombosis and myocardial infarction (1). For these patients, systemic anticoagulation is typically recommended in addition to antiplatelet therapy (3). Historically, pediatric thrombosis prevention relied on heparin, LMWH, and VKAs; although effective, these agents pose significant challenges and limitations, including frequent laboratory monitoring, injection use, and drug–food interactions (49, 50). Recognizing these challenges, the 2024 American Heart Association statement on KD acknowledges DOACs as a promising alternative anticoagulation strategy in select patients with large and giant CAAs who require long-term treatment (3). These guidelines highlight the appeal of DOACs as an alternative therapy due to their predictability, oral administration, and reduced monitoring requirements compared with their traditional counterparts.

The US FDA approved the use of rivaroxaban, a direct factor Xa inhibitor, for pediatric venous thromboembolism (VTE) following the phase 3 EINSTEIN-Jr randomized trial (51). In this multicenter study conducted across 107 pediatric hospitals, 500 pediatric patients with acute VTE were assigned to receive either bodyweight-adjusted rivaroxaban or standard anticoagulants, including heparin or VKAs. The primary efficacy outcome was recurrent symptomatic VTE, while safety outcomes included major and clinically relevant non-major bleeding. After a median follow-up of 91 days, recurrent VTE occurred in 1% of children receiving rivaroxaban compared with 3% of those receiving standard anticoagulants (HR 0.40, 95% CI 0.11–1.41). In addition, thrombotic burden was shown to be decreased in the rivaroxaban group than in the standard anticoagulation group on repeat imaging (*P* = 0.012). The incidence of major bleeding was low and similar between the two groups, and this study established rivaroxaban as a safe and effective alternative for traditional anticoagulants in pediatric patients (51). The findings of the EINSTEIN-Jr trial have laid the foundation for studies in patients with Kawasaki disease, as is the case with an ongoing RCT evaluating rivaroxaban in children older than 2 years with giant CAAs following a diagnosis of Kawasaki disease (52).

In the context of KD, the DOAC edoxaban, which has the same mechanism of action as rivaroxaban, also shows potential for antithrombotic activity in the treatment of KD. Sagiv et al. conducted a retrospective study using data from 16 pediatric patients who received edoxaban at Seattle Children's Hospital between 2018 and 2022 (53). All patients had a history of KD with evidence of coronary aneurysms at the time of active symptoms, alongside one patient with coronary aneurysms secondary to antineutrophil cytoplasmic autoantibody-associated vasculitis, which the investigators considered comparable to KD vasculitis. When edoxaban was used in conjunction with low-dose aspirin (3–5 mg/kg/day), the study found that 84% of participants experienced no thrombotic events at 48 months (53). Considerable limitations of this study include the absence of a control group, such as an aspirin-only group, for direct comparison and the lack of specification of the percentage of participants free from thrombotic events at 4 years following the initiation of edoxaban. Similar to the clopidogrel-vs.-aspirin study (42), this research suffered from low statistical power due to an extremely small sample size (*n* = 16), which limits its generalizability.

Dabigatran, a direct thrombin inhibitor, has substantial evidence supporting its use for the secondary prevention and treatment of VTE. The phase 2b/3 DIVERSITY trial was a randomized, controlled, open-label, non-inferiority trial comparing dabigatran with standard anticoagulants, such as LMWH, unfractionated heparin, VKAs, or fondaparinux, in children younger than 18 years with acute VTE who were initially treated with parental anticoagulation and required an additional 3 months of anticoagulation therapy. The study showed that dabigatran was non-inferior to standard VTE treatment in efficacy and successfully established an age- and weight-adjusted dosing algorithm, ultimately leading to FDA approval for dabigatran use in pediatric populations with VTE (54).

Overall, previous studies and trials suggest that DOACs may serve as a promising adjunctive therapy in the treatment of KD patients with giant CAAs. However, it must be emphasized that anticoagulants such as DOACs and direct thrombin inhibitors are primarily used for the prevention of venous thromboembolism. KD is characterized by arterial thrombosis and coronary aneurysms rather than venous pathology. Despite this difference in vasculature, antiplatelet and anticoagulant drugs are used in combination for various diseases, including the acute management of acute coronary syndrome. Anticoagulants could be used in conjunction with antiplatelet agents in KD. However, this idea is theoretical, and a series of RCTs is required to ensure sufficient safety and efficacy before considering this drug regimen in the setting of KD. Therefore, antiplatelet agents and IVIG should remain the primary foundation of KD treatment rather than anticoagulant agents.

## 4 Conclusions

Therefore, with the utility of aspirin questioned by the proper use of IVIG and the potential of steroids to quell inflammation, this paper calls for further investigation into the role of aspirin in the management of KD patients. However, the anti-inflammatory properties of aspirin must be recognized, as they may play a vital role in preventing the development of coronary aneurysms, while its antiplatelet capabilities limit clot formation if aneurysms do occur. Furthermore, case reports of Reye syndrome associated with aspirin therapy for KD occurred mostly in the setting of using high-dose aspirin. Perhaps the use of medium- or low-dose aspirin may provide the necessary treatment for KD without the risk of Reye syndrome. Therefore, this review recognizes that aspirin remains a primary component of standard KD therapy and has contributed to the successful treatment of countless patients. This review also notes how recent literature suggests that IVIG, corticosteroids, or their combination can provide the anti-inflammatory therapy necessary to minimize coronary artery abnormalities. However, because aspirin offers the second benefit of acting as an antiplatelet drug, further research should aim to distinguish the effectiveness of both the anti-inflammatory and antiplatelet capabilities of aspirin compared with IVIG, corticosteroids, or their combination. While conducting RCTs remains challenging due to the rarity of KD, further research is needed among pediatric rather than adult populations to determine whether modifications to current pharmaceutical treatment are needed. However, until further research establishes the definitive safety of potential alternative therapies, this paper recommends the continued management of KD according to established clinical guidelines, with treatment centered on aspirin and IVIG, despite the remarkably rare phenomenon of Reye syndrome. Looking forward, the complex cascade of platelet activation and aggregation, coupled with a broad selection of medications, offers abundant avenues for future research.
